# Variation in resistance force during intraocular lenses injection by modern injectors and assessment of damage to the injector: a laboratory analysis

**DOI:** 10.1038/s41598-026-41145-7

**Published:** 2026-05-06

**Authors:** Maximilian Friedrich, Victor A. Augustin, Donald J. Munro, Maximilian Hammer, Sonja Schickhardt, Hyeck-Soo Son, Gerd U. Auffarth

**Affiliations:** The David J. Apple® Center for Vision Research, Department of Ophthalmology, University of Heidelberg, Im Neuenheimer Feld 400, 69120 Heidelberg Baden-Wuerttemberg, Germany

**Keywords:** Resistance force, Injector system, Intraocular lens, Area under the curve, Diseases, Health care, Medical research

## Abstract

This experimental study aimed to guide cataract surgeons in selecting suitable intraocular lens (IOL) injectors by evaluating the in vitro resistance force during IOL ejection and assessing associated injector damage across modern injector models. Twenty-five injectors from five manufacturers were tested using + 20 diopter lenses. IOLs were ejected into petri dishes, and resistance forces were continuously recorded with an automated digital force gauge. Parameters including maximum force and the area under the curve (AUC) were analyzed. After each ejection, injector nozzle tips were examined microscopically and graded for damage using the HeiScore. Significant differences in resistance forces were found among injector models (*p* < 0.05), with maximum forces ranging from 4.39 ± 0.45 N to 25.71 ± 6.62 N. The AUC also showed significant variability, indicating differing levels of force required for IOL delivery. No ejection complications occurred, though one injector model exhibited markedly higher nozzle damage compared to others. These findings demonstrate substantial variability in resistance and durability among modern IOL injectors and underscore the importance of understanding these differences to ensure safe, controlled, and efficient IOL implantation in cataract surgery.

## Introduction

In modern cataract surgery, intraocular lenses (IOLs) are implanted in the eye with an IOL injector. Using an injector reduces incision size compared with implanting IOLs held by forceps, thereby reducing surgically induced astigmatism^1^. A variety of injectors are currently available from many manufacturers, each differing in its design, handling, and the required incision size^2–4^.

Ten years ago, a laboratory study in Japan evaluated the resistance force measurements of several IOL injectors that were available at the time^5^. However, most of the injectors that were examined by Usui and Tanaka have become obsolete, due to advances in both the design of IOLs and of their injectors. We set out to analyze the resistance force profiles of modern IOL injectors and compare their ease of use.

Previously, our study group published the HeiScore scale to assess damage to the injector tip during clinical use^4,6^. Damage profiles varied significantly across injectors. Some can exhibit a bursting injector tip during IOL implantation, potentially leading to intraocular foreign bodies. There is a need for an independent, objective assessment of commonly available injectors to provide transparent information to cataract surgeons.

The objective of this study was to compare the resistance force profiles and complications of five different widely available IOL injectors during IOL ejection.

## Methods

### Study materials

In this exploratory laboratory analysis, five injectors from different manufacturers were included as shown in Table 1. Four of the five were preloaded injectors, where the manufacturer preloaded its own lens model, thus the lens and its injector were from the same manufacturer. For one of the five injector models, we manually loaded a Teleon lens in a Medicel Viscoject-BIO 2.2, the injector model recommended by Teleon. All lenses had a labelled power of + 20.0 D. This study did not need an ethics committee approval as no experiments on humans or animals were performed.

**Table 1:** Summary of the studied intraocular lenses and injector models.

Lens and injector combination	Manufacturer(s)	Lens power (Diopter)	IOL material	IOL loading
Avansee Preload1P YP2.2 V	Kowa Co.	+ 20.0	Hydrophobic	Preloaded
CT Lucia 621P	Zeiss AG	+ 20.0	Hydrophobic	Preloaded
RayOne Hydrophobic Aspheric RAO800C	Rayner Ltd.	+ 20.0	Hydrophobic	Preloaded
Teleon Lentis Quantum + Viscoject-BIO 2.2	Teleon BV + Medicel AG	+ 20.0	Hydrophilic with hydrophobic surface	Manually loaded
Vivinex multiSert XC1-SP	Hoya Co.	+ 20.0	Hydrophobic	Preloaded

### Surgical procedure

The experiments were conducted in a laboratory at a temperature of 23 °C and 50% relative humidity, which matches the setting in an operating room, which is also approximately 23 °C.

All injectors were prepared according to the manufacturer’s instructions, using a sodium hyaluronate ophthalmic viscosurgical device (OVD) as a lubricant. All IOLs were ejected into a petri dish and the push force needed to eject the lens was measured using an automated force meter (Shimpo Check-line FGV-XY digital force gauge) with the Shimpo Toriemon Force Gauge Software. All IOL ejections were performed by a single surgeon (MF). Changes in the resistance force are shown as a curve in a graph, and the maximum peak of each curve in the graph was evaluated. The size of the total area under the curve (AUC) was determined using numerical integration. After ejection of the IOL from the injector, the injector nozzle tips were examined by optical microscopy (Olympus BX50 microscope (Olympus) with an attached Olympus Camedia C-7070 Wide Zoom camera (Olympus)) and the observable damage was graded according to the HeiScore first described by Fang et al.^6^.

### Statistical analysis

Using R statistical software (Version 4.2.2, R Foundation for Statistical Computing, Vienna, Austria), a Kruskal-Wallis test was performed to analyze whether there are significant differences between all groups in terms of the maximum resistance force and the area under the curve of the resistance force. If the Kruskal-Wallis test was significant, a pairwise Wilcoxon test was performed. For the Kruskal-Wallis test a P-value < 0.05 was considered significant. For the subgroup analysis, the P-values were adjusted due to the performance of multiple significant tests according to the Bonferroni-method.

## Results

**Fig. 1:**
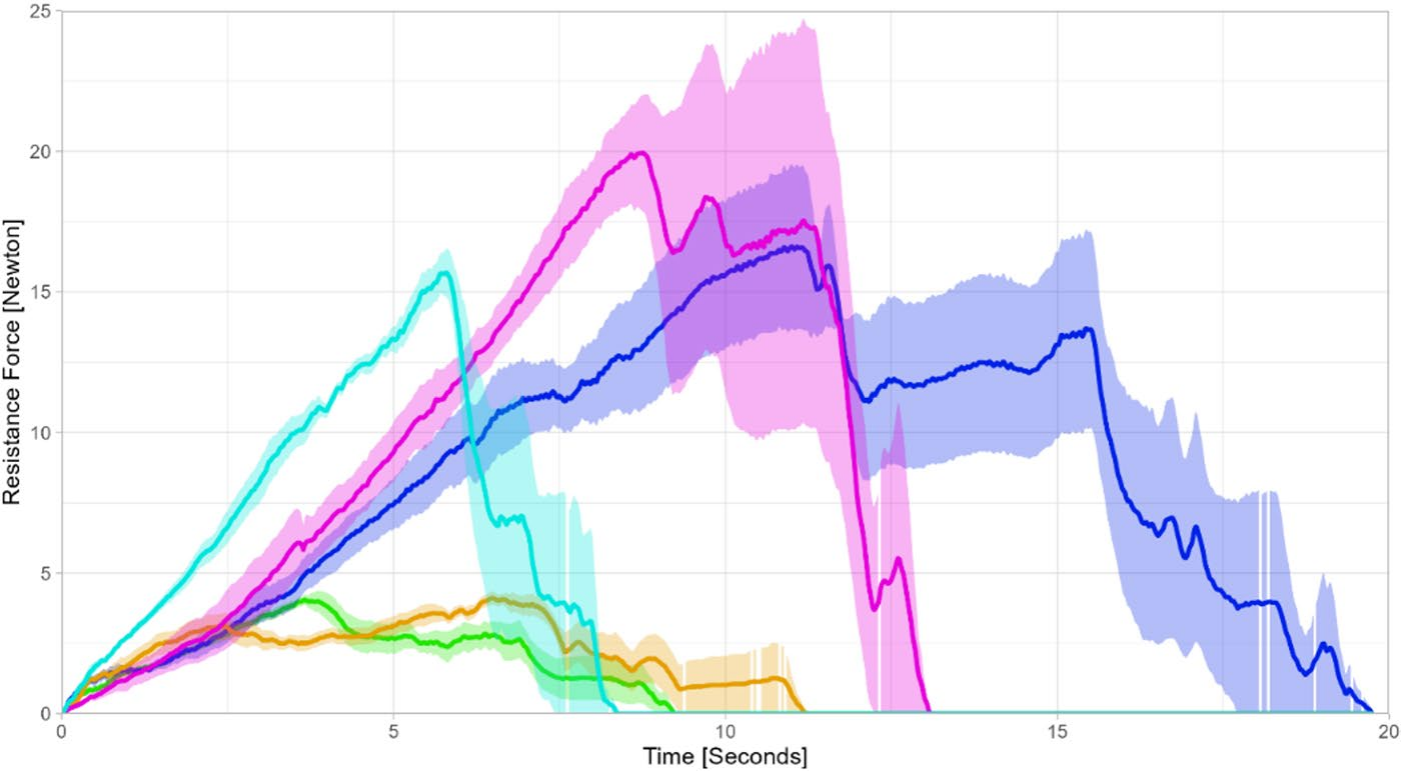
Overview of the mean resistance force over time during intraocular lens ejection depending on injector (*n* = 5 for each model). Shaded area: Standard error. Orange: Kowa Avansee. Blue: Zeiss Lucia. Purple: Rayner RayOne. Turquoise: Teleon Lentis Quantum & Viscoject-BIO. Green: Hoya multiSert.

The resistance force profiles differed significantly depending on which injector was used, as shown in Fig. 1. The resistance force profiles of the Kowa Avansee Preload1P injector system and the Hoya multiSert Vivinex XC1-SP showed a flat curve enabling a smooth and controlled IOL ejection without resistance spikes. The force profiles of the Zeiss CT Lucia 621P, the Rayner RayOne Hydrophobic Aspheric, and the Teleon Lentis Quantum with the Viscoject-BIO 2.2 showed a steeper increase in resistance force with a clear force peak and a fast decline after IOL ejection.

**Fig. 2:**
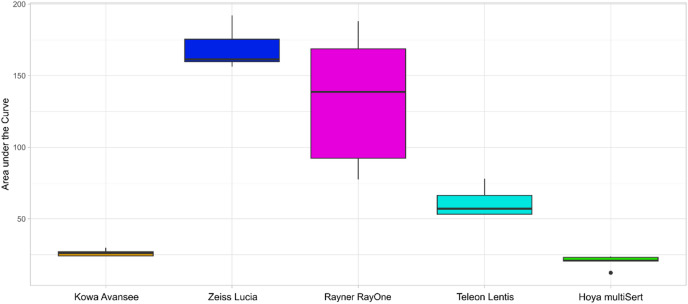
Area under the curve of the resistance force during intraocular lens ejection depending on injector (*n* = 5 for each model).

**Fig. 3:**
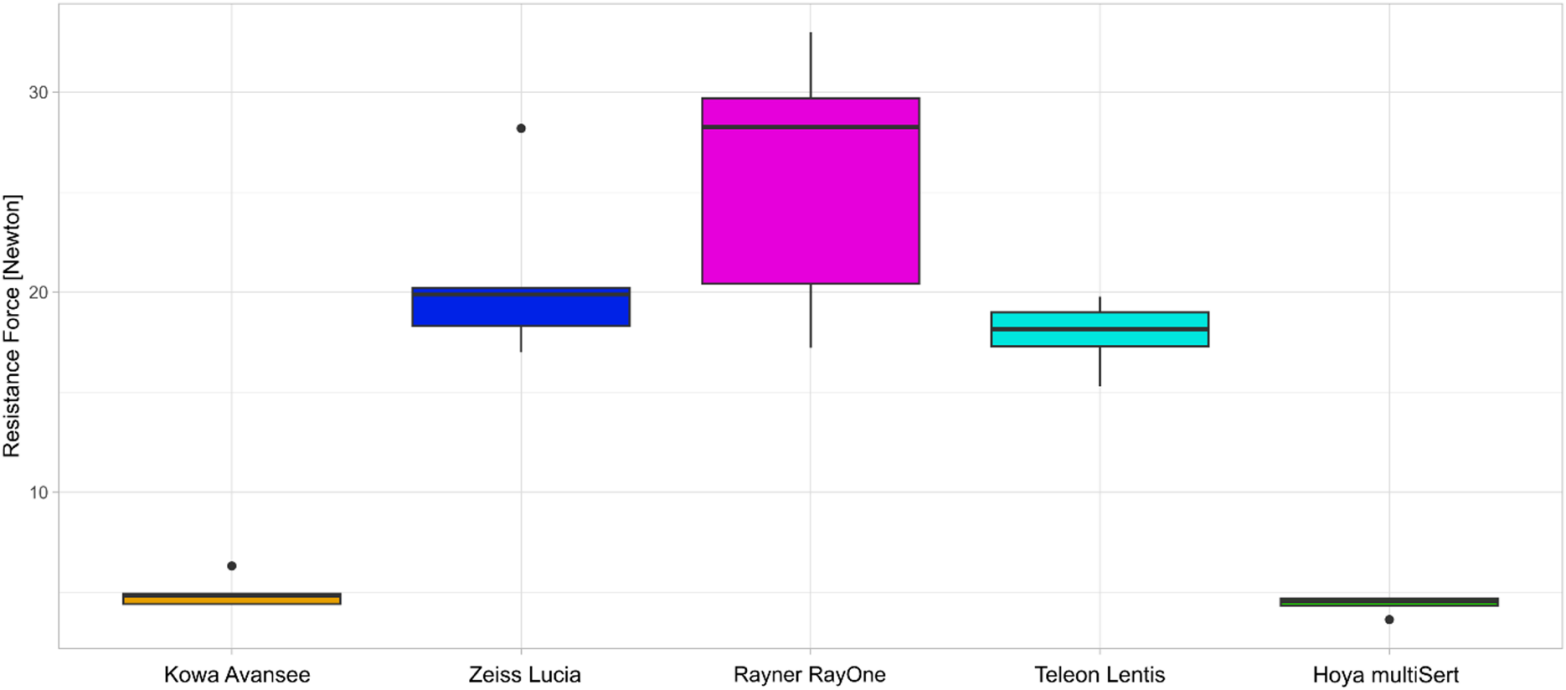
Maximum resistance force during intraocular lens ejection depending on injector (*n* = 5 for each model).

The area under the curve of the resistance force curves and maximum resistance force values during the ejection process are shown in Figs. 2 and 3, respectively. The Kruskal-Wallis test showed statistically significant differences between the groups regarding the area under the curve (*p* < 0.001) and the maximum resistance force (*p* < 0.001).

The area under the curve of the Hoya multiSert Vivinex XC1-SP was significantly lower than the Kowa Avansee Preload1P injector system (*p* = 0.009) as well as lower than all other injectors (*p* < 0.001). The Kowa Avansee Preload1P injector system showed a significantly lower area under the curve than the Zeiss CT Lucia 621P (*p* = 0.009), the Rayner RayOne Hydrophobic Aspheric (*p* = 0.009), the Teleon Lentis Quantum with the Viscoject-BIO 2.2 (*p* = 0.009).

The Kowa Avansee Preload1P injector system and Hoya multiSert showed a significantly lower maximum resistance force than the Zeiss CT Lucia 621P (*p* = 0.009), the Rayner RayOne Hydrophobic Aspheric (*p* = 0.009), the Teleon Lentis Quantum with the Viscoject-BIO 2.2 (*p* = 0.009). There was no significant difference between the Kowa Avansee Preload1P injector system to the Hoya multiSert (*p* > 0.05).

**Fig. 4:**
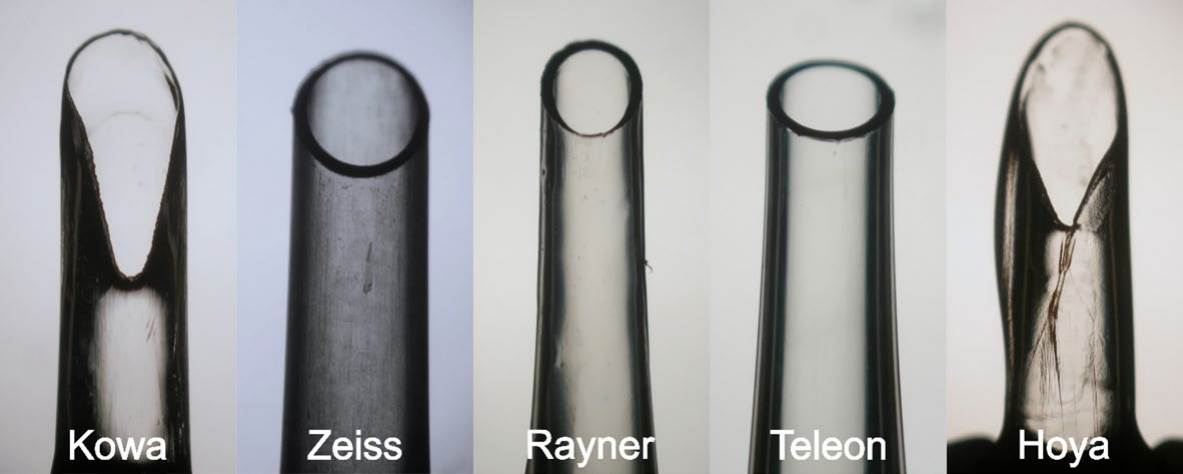
Example of microscopical postoperative HeiScore grading of injector nozzles.

The injector damage HeiScores are summarized in Table 2. Four of the injector models showed a low damage profile with only minor superficial scratches at the injector tip. In contrast, all five Hoya MultiSert injectors showed a full thickness crack of the injector tip, depicted in Fig. 4.

**Table 2:** HeiScore values for the studied injectors: nozzle damage after injection.

Lens and injector combination	Manufacturer (s)	Median	Minimum	Maximum
Avansee Preload1P YP2.2 V	Kowa Co.	0	0	1
CT Lucia 621P	Zeiss AG	0	0	1
RayOne Hydrophobic Aspheric RAO800C	Rayner Ltd.	0	0	1
Teleon Lentis Quantum + Viscoject-BIO 2.2	Teleon BV + M edicel AG	1	0	1
Vivinex multiSert XC1-SP	Hoya Co.	4	4	4

## Discussion

In this laboratory study we compared the resistance force profiles and injector tip damage of five commonly available IOL injectors. The results demonstrated significant differences between injectors regarding maximum resistance force, the area under the resistance force curve, and the morphology of the respective resistance force profile. Injectors such as the Kowa Avansee Preload1P and the Hoya multiSert Vivinex XC1-SP showed flatter resistance force curves with lower maximum values, while the Zeiss CT Lucia 621P, Rayner RayOne Hydrophobic Aspheric, and Teleon Lentis Quantum with the Viscoject-BIO 2.2 showed steeper inclines and more pronounced force peaks. These findings suggest that injector design continues to significantly influence IOL injection dynamics. Importantly, while such differences in force characteristics may influence the subjective perception of controllability, no definitive resistance force threshold associated with intraoperative complications has been established to date. In this context, maximum resistance force may be considered a measure of transient peak loading, whereas the area under the resistance force curve reflects the cumulative resistance encountered throughout the implantation process and may better represent sustained handling characteristics.

Our results align with the earlier work of Usui and Tanaka, who reported considerable variability in resistance force values between injectors that were available more than a decade ago^5^. They had found that two injectors needed a significantly higher force to implant the IOL than three other injector models. Ten years later, we still found that three of the five models we studied exhibit steep, high-resistance force profiles compared to their competitors, underscoring that injector performance is not uniform across contemporary systems. Usui and Tanaka also found that resistance forces are similar during IOL injections into a petri dish and into a porcine eye, suggesting that resistance force depends on injector parameters and is independent of ocular anatomy and incisional wound structures.

In addition to force measurements, we evaluated nozzle damage using the HeiScore as introduced by Fang et al.^6^ That work demonstrated that injector generations differ considerably with respect to structural integrity of the tip after IOL ejection, and highlighted the potential for tip rupture. In our analysis, most modern systems showed only minor superficial damage. The exception was the Hoya multiSert, which consistently exhibited full-thickness cracks at the nozzle tip as reported previously^4,7^. Notably, this occurred despite comparatively low resistance force values, suggesting that resistance force magnitude alone does not fully capture the mechanical stresses acting on the injector nozzle. A possible explanation may lie in injector-specific factors such as nozzle geometry, material composition, and local stress concentration during IOL passage, which may predispose to structural damage even in the absence of high overall resistance forces. Zhang et al. compared three injectors with a V-shaped nozzle tip including the Hoya multiSert and the older Kowa Avansee injector system^7^. They also found that the multiSert exhibited more nozzle damage than the Kowa Avansee injector system, suggesting that a V-shaped nozzle tip alone does not necessarily indicate more propensity to become damaged. While these findings were observed in vitro, such damage could theoretically release fragments or foreign material into the anterior chamber, emphasizing the importance of device-specific evaluation. However, the findings in vitro were similar to a prospective randomized comparative in vivo study by Baur et al., which reported a HeiScore of the Hoya multiSert of 3.68 ± 0.47^8^. In addition, Zhang et al.,^9^ found in a clinical study that the median HeiScore value of the RayOne injector system was “1”, which is similar to our results.

From a clinical perspective, injectors with lower peak forces and smoother resistance profiles may allow more predictable and controlled IOL delivery and potentially reduce the risk of sudden IOL propulsion or wound stress during IOL insertion. However, higher resistance forces do not necessarily imply abrupt or uncontrolled IOL propulsion and may also be encountered during a smooth and continuous implantation process, depending on injector design and surgical handling. Nevertheless, increased resistance may be perceived as reduced controllability, which under certain circumstances might contribute to sudden IOL propulsion during IOL insertion. Conversely, devices with steep force profiles or evidence of structural compromise may carry a higher risk of intraoperative challenges. These implications remain speculative, as this study was conducted under laboratory conditions, but they underscore the need for transparent comparative data to inform surgeons’ choice of injector systems.

Several limitations of the present study should be acknowledged. First, this was an exploratory laboratory analysis, which does not fully replicate the in vivo surgical environment where factors such as incision size, anatomy of the anterior chamber, and various perioperative conditions may influence injector performance. Second, only one IOL power (+ 20.0 D) was examined and thus our observation cannot be generalized for other IOL powers. In particular, higher-power IOLs, which are typically thicker, may exhibit different resistance characteristics and may interact differently with specific injector designs. The influence of IOL power on resistance forces and controllability should be addressed in future studies. Third, only hydrophobic or hydrophobic-hybrid IOLs were tested. Therefore, our findings may not apply to purely hydrophilic lenses or to lenses with different optic or haptic designs. Finally, one of the evaluated injector systems required manual loading, whereas the others were preloaded. Although we consider resistance forces to be primarily determined by injector nozzle geometry and IOL material properties rather than by the loading method itself, the use of a manually loaded system represents a methodological difference that should be taken into account when interpreting inter-system comparisons.

Nonetheless, our results show that modern IOL injectors display distinct resistance force profiles and differ in postoperative nozzle damage. Some devices allow smoother, lower-resistance IOL delivery, while others show higher force peaks or structural vulnerability of the injector tip. These differences should be recognized by surgeons, to prevent injector-related complications intraoperatively. Further improvements in injector material and design may improve the resistance profile to allow a smooth and safe IOL implantation without relevant damage to the injector nozzle or the operated eye.

## Data Availability

The datasets generated during and/or analyzed during the current study are available from the corresponding author on reasonable request.

## Abbreviation

IOL: Intraocular lens
